# Legacy in Cardiovascular Risk Factors Control: From Theory to Future Therapeutic Strategies?

**DOI:** 10.3390/antiox10111849

**Published:** 2021-11-22

**Authors:** Lucie Pothen, Jean-Luc Balligand

**Affiliations:** Institute of Experimental and Clinical Research (IREC), Pharmacology and Therapeutics (FATH), Cliniques Universitaires St-Luc and Université Catholique de Louvain (UCLouvain), 1200 Brussels, Belgium; lucie.pothen@uclouvain.be

**Keywords:** metabolic memory, legacy effect, ROS, diabetes, hypertension, angiotensin II, oxLDL, hypercholesterolemia

## Abstract

In medicine, a legacy effect is defined as the sustained beneficial effect of a given treatment on disease outcomes, even after cessation of the intervention. Initially described in optimized control of diabetes, it was also observed in clinical trials exploring intensification strategies for other cardiovascular risk factors, such as hypertension or hypercholesterolemia. Mechanisms of legacy were particularly deciphered in diabetes, leading to the concept of metabolic memory. In a more discreet manner, other memory phenomena were also described in preclinical studies that demonstrated long-lasting deleterious effects of lipids or angiotensin II on vascular wall components. Interestingly, epigenetic changes and reactive oxygen species (ROS) appear to be common features of “memory” of the vascular wall.

## 1. Introduction

Cardiovascular diseases (CVDs) are the leading cause of death worldwide, with a major impact not only in developed nations but also in low- and middle-income countries, where they account for nearly 30 percent of all deaths [1]. Risk factors are well known: diabetes, hypercholesterolemia, smoking, hypertension, obesity, aging, familial history of early cardiovascular diseases, or sedentary lifestyle. Control of modifiable cardiovascular risk factors is essential to reduce the incidence of CVD, especially at early stages, before vascular damage develops [2,3]. Legacy in English refers to what one generation passes to the next or something that remains from an earlier time. In medicine, a legacy effect is defined as the sustained beneficial effect of a given treatment on disease outcomes or complications, even after cessation of the intervention. The term was initially used in the context of diabetes when long-term follow-up results of two diabetes management trials—the Diabetes Control and Complications Trial (DCCT) and the United Kingdom Prospective Diabetes Study (UKPDS)—were revealed [4,5]. A similar lasting effect was observed in lipid-lowering and blood pressure control trials [6].

**Figure 1:**
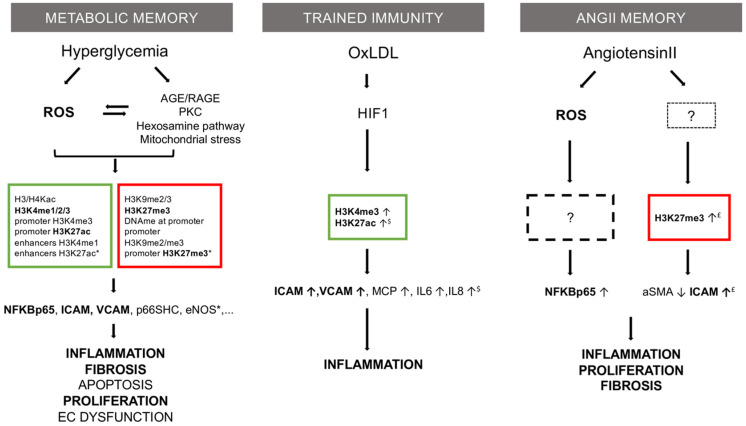
Mechanistic comparison of three memory theories in vascular research: metabolic memory, trained immunity, and Ang II memory. Common features are in bold type. Described epigenetic marks are listed in squares (red = repressive; green = permissive). Based on: * [7], ^$^ [8] and ^£^ [Pothen et al., unpublished data].

In this paper, we reviewed clinical trials that revealed a legacy effect and established this concept in cardiovascular medicine. We searched in PubMed MEDLINE database for English written studies using specific terms: “legacy effect”, “metabolic memory”, “diabetes”, “hypertension”, and “hypercholesterolemia”. A vast majority of included trials were randomized control trials, with an open label follow-up period (Table 1). In parallel, we summarized key data from basic research pointing to potential pathophysiological mechanism of legacy effects, based on previous studies on metabolic memory (Figure 1).

## 2. Diabetes

### 2.1. Legacy Effect in Diabetic Patients

Historically, the concept of the legacy effect emerged at the beginning of 21th century from two clinical studies conducted in diabetic patients (Table 1). The DCCT study was evaluating the effect of intensive control of glycemia compared to conventional therapy in a cohort of 1441 type 1 diabetic patients. After more than 6 years of intervention, the development of microvascular complications was significantly diminished in the intensive treatment group, i.e., severe retinopathy (by 53%), clinical neuropathy (by 40%), and microalbuminuria (by 61%) [4]. After the trial, patients were followed for 17 years in the Epidemiology of Diabetes Interventions and Complications (EDIC) study. Despite interruption of the intensive control on glycemia (7.9% of Hb1Ac in previously intensive treatment group; 7.8% in conventional group), the authors observed a persistent reduction in the incidence of cardiovascular events (nonfatal MI, stroke, or death from CVD of 57%) and nephropathy (of 46%) in the former intensive treatment group [9].

Concordant results were observed later in UKPDS, a study conducted in 3867 newly diagnosed type 2 diabetic patients. In the intervention period, after a three-month diet, patients were randomized either to intensive glucose-lowering treatment (sulfonylurea, insulin, metformin) or to conventional dietary management and followed up for 10 years. Intensive glucose lowering was associated with a significant decrease in any diabetes-related endpoint (of 12%), any diabetes-related death (of 10%), and microvascular endpoints (of 25%). At this point, there was no significant reduction in macrovascular endpoints [5]. Another 10 years of follow-up of this cohort showed persistent benefit despite no difference in HbA1c already after 1 year. In the former intensively treated group, the relative risk reductions persisted for any diabetes-related endpoint (by 9%) and for microvascular disease (24%) and were extended also for MI (by 15%) and for death from any cause (13%) [10].

Subsequently, several other studies failed to reproduce the same legacy effect: the Veterans Affairs Diabetes Trial (VADT) performed in long-standing type 2 diabetic patients [11,12,13], the Action to Control Cardiovascular Risk in Diabetes (ACCORD) trial with more than 10,000 long-lasting type 2 diabetic patients [14,15], or the Action in Diabetes and Vascular Disease Preterax and Diamicron (ADVANCE) study in 11,140 type 2 diabetic patients [16,17]. Long-term follow-up did not reveal any long-term benefit of the intervention period in all these cohorts. A common feature of these randomized trials was the inclusion of long-lasting diabetic patients (around 10 years of disease) and, in some studies, a high proportion of patients who already presented one cardiovascular event at randomization (e.g., VADT).

On the other hand, additional real-life studies reinforced the legacy effect theory in diabetes control, such as the Fremantle Diabetes Study [18], the Anglo–Danish-Dutch Study of Intensive Treatment in People with Screen-Detected Diabetes in Primary Care (ADDITION) [19,20], and the Diabetes and Aging Study (DAS) [21].

Taken together, the above-mentioned data suggest that to obtain a legacy effect, interventions on glycemic control need to be implemented early in the disease, certainly before any cardiovascular event, perhaps even within the first year. The accrued evidence then led to a new concept in diabetes clinical care: the sooner, the better.

Conversely, the failure to obtain a favorable effect from intensive treatment in long-lasting, under-controlled diabetic patients could be ascribed to a “metabolic memory” underlying an adverse legacy effect, as explained below.

### 2.2. “Metabolic Memory”: What Is Hidden behind the Legacy Effect

The phenomenon of “metabolic memory” had already been described in animal models before the concept of the legacy effect emerged from clinical studies. In 1987, in a dog model of diabetic retinopathy, Engerman et al. observed that animals with late onset optimization of glycemic control had a higher incidence of progression of this microvascular complication of diabetes [22]. A few years later, the word “memory” appeared in the scientific literature with the observation of lasting overexpression of fibronectin mRNA in several organs (e.g., kidney and heart) of streptozotocin-induced diabetic rats, despite restoration of euglycemia. The same authors observed overexpression of fibronectin and collagen IV in cultured endothelial cells exposed to hyperglycemia. This increased expression was sustained in time despite normalization of glucose concentration in culture media and subsequent cell passages [23]. The observation that early—but not late—islet transplantation, i.e., before retinal changes, prevented progression of retinopathy in sucrose-fed diabetic Cohen rats further substantiated the memory theory [24]. Metabolic memory was further illustrated in several models in vitro and in vivo, including endothelial or vascular smooth muscle cells in culture, but also in animal models of atherosclerosis, diabetic nephropathy, and retinopathy [25,26].

These models allowed the proposal of some putative mechanisms as follows: hyperglycemia induces an overproduction of mitochondrial superoxide. The ensuing oxidative stress leads to the activation of five major pathways: increased formation of AGEs (advanced glycation end products), expression of its receptor (RAGE), activation of protein kinase C isoforms, overactivity of the hexosamine pathway and polyol pathway that, in turn, activate inflammatory reactions. The same mediators also produce long-lasting epigenetic changes driving sustained expression of proinflammatory genes, despite restoration of glycemic control, leading to the observed hyperglycemic memory [27,28,29,30] (Figure 1). As an example, several histone lysine methylations were described following transient high glucose levels, associated with persistent transcriptional induction of the RELA gene, coding for the p65 subunit of NF-kB, despite subsequent incubation of cells with normal glucose concentrations; this led to transcriptional activation of target genes implicated in inflammation and endothelial dysfunction [31]. Indeed, hyperglycemia-dependent ROS production induced mono-methylation of histone 3 at lysine 4 amino-acid residue (H3K4me) by the methyltransferase Set7/9, a post-translational modification favoring gene transcription in mammals; the ensuing modification of histone binding the proximal promoter region of the RELA gene resulted in upregulation of the NF-κB subunit p65 and persistent vascular inflammation [29]. ROS production also induced CpG hypomethylation and increased H3 histone acetylation in the p66Shc promoter region; this mitochondrial adaptor, in turn, modulated the intracellular redox state, so ROS-induced epigenetic modifications were associated with higher levels of p66Shc and with significant activation of PKC, therefore sustaining oxidative stress, endothelial dysfunction, and vascular damages [32,33].

Moreover, upon epigenome profiling of white blood cells of patients from the DCCT trial, an enrichment of histone acetylation marks as promoters of inflammatory genes and other genes related to diabetic complications was observed in selected patients with progressive retinopathy and nephropathy compared to controls. This epigenetic mark also significantly correlated with the mean HbA1C during the trial [34].

## 3. Hypercholesterolemia

### 3.1. Legacy Effect from Optimization of Lipid Profile

As for diabetic patients and optimized glycemic control, lipid-lowering therapies also showed long-term benefit on cardiovascular outcomes (Table 1). In the Long-term Intervention with Pravastatin in Ischaemic Disease (LIPID) trial, pravastatin given in secondary prevention for 6 years reduced mortality and cardiovascular events in patients with previous acute coronary syndromes [35]. After a subsequent open-label period of 2 years, in which patients were offered pravastatin irrespective of their original assigned therapy, a sustained significant benefit of the initial intervention was observed on all-cause mortality, coronary heart disease mortality, myocardial infarction, and stroke [36].

In the Anglo-Scandinavian Cardiac Outcomes Trial—Lipid Lowering Arm (ASCOT-LLA), 10,000 hypercholesterolemic and hypertensive patients with at least three other cardiovascular risk factors were randomized to receive either atorvastatin or placebo in primary prevention. The trial was stopped prematurely (median of 3.3 years of intervention) because of a 36% relative risk reduction in the primary outcome (consisting of non-fatal myocardial infarction and fatal coronary heart disease) in favor of atorvastatin and a non-significant reduction in CV deaths (of 16%) and all-cause mortality (of 13%) [37]. In the long-term follow-up (median of 11 years), all-cause mortality appeared significantly lower in patients originally assigned atorvastatin (decrease of 14%). Fewer cardiovascular deaths were observed (11% less; not significant), as well as non-cardiovascular deaths (15%; reaching significance), in those formerly assigned atorvastatin, despite extensive crossover to statin treatment after the end of the initial trial. This reduction was intriguingly driven by a reduction in deaths due to infection and respiratory illness, raising still the unanswered question of the underlying mechanisms [38]. In the West of Scotland Coronary Prevention Study (WOSCOPS), men were proposed pravastatin as primary prevention or placebo for 5 years. Once again, on-trial statin treatment produced a beneficial effect vs. placebo on nonfatal myocardial infarctions (by 31%), death from coronary heart disease (by 28%), and death from all cardiovascular causes (by 32%), despite low adherence at this point (only 38.7% of the intervention group were found to be on statins at 5 years) [39]. More than 20 years of follow-up revealed a sustained beneficial effect on all-cause mortality (by 13%), attributable mainly to a 21% decrease in cardiovascular death [40], again despite crossover to statins in the original placebo group after the trial. The above-mentioned study ACCORD included a lipid arm (ACCORD-Lipid), in which type 2 diabetic patients were randomized to simvastatin plus fibrate or simvastatin plus placebo for 5 years. At the end of the intervention, there was no evidence of a beneficial effect of combined therapy compared to statins alone on cardiovascular outcomes and mortality [41]. However, the follow-up study (ACCORDION) showed that allocation to the combined fibrate–statin treatment arm during the trial period had a beneficial legacy effect on all-cause mortality (35%), on top of decreased incidence rates of non-fatal myocardial infarction, congestive heart failure, major coronary heart disease, and CVD mortality [42]. The Heart Outcomes Prevention Evaluation (HOPE)-3 trial evaluated the benefit of lipid-lowering therapy (using rosuvastatin) and/or blood pressure optimization (using candesartan plus hydrochlorothiazide) in 12,000 intermediate-risk patients, e.g., patients with at least one cardiovascular risk factor, without any previous cardiovascular event. To note, inclusion in the trial did not mandate specific lipid or blood pressure levels before randomization. Each intervention was compared to placebo (the study included also combined treatment) for a duration of almost 6 years. Treatment with rosuvastatin was associated with a significant decrease (of 24%) for the first coprimary outcome, namely MACE-1 (composite of death from CV causes, non-fatal myocardial infraction, non-fatal stroke), and consistently, with a significant decrease (of 25%) for the second outcome, namely MACE-2 (composite of revascularization, heart failure, resuscitated cardiac arrest) [43]. In the recently published analysis of the long-term follow-up of 8.7 years, the benefit of rosuvastatin was maintained with a 21% reduction in risk of MACE-1 and a 21% reduction in risk of MACE-2 for the total follow-up. To note, only 36% of patients initially randomized to rosuvastatin and 38% of those randomized to placebo were prescribed a statin after the active phase of the study [44]. A recent meta-analysis evaluated the legacy effect of lipid-lowering treatment by statins, analyzing eight randomized studies, either in primary or secondary prevention. Globally, direct in-trial effects were greater than post-trial effects (likely attributable to crossover effects), regarding cardiovascular and all-cause mortality. There was no evidence of significant legacy effect on CVD mortality, but some evidence of legacy effect on all-cause mortality; a subgroup analysis of the three trials performed as a primary prevention also demonstrated legacy effect on both CVD mortality (by 13%) and all-cause mortality (by 10%) [45].

The Antihypertensive and Lipid-Lowering Treatment to Prevent Heart Attack Trial (ALLHAT) included a subgroup in which hypertensive patients with a least another cardiovascular risk factor received pravastatin compared to usual care. No effect of the intervention was observed in all-cause mortality or coronary heart disease. This may be due to the modest differential in lipid profile between the intervention group and usual care group (16.7% lower LDL-C compared to control). Moreover, the study was not blinded, at a period of publication of a series of guidelines stimulating optimization of the lipid profile and statin use contributed to treatment crossovers. Indeed, the trial follow-up showed a decrease in total cholesterol and LDL-C over time in both groups [46]. Consistently, long-term follow-up did not reveal any legacy effect of pravastatin in this trial [47]. The Second Australian National BP Study (ANBP2) was an open-label study including older hypertensive patients that was designed to compare two anti-hypertensive therapies. In a post hoc analysis of the ANBP2 cohort, patients were stratified according to lipid-lowering therapy at entry as the primary prevention versus usual care. Outcomes were compared regardless of their randomized treatment. Lipid-lowering treatment was significantly associated with a long-term (11 years) decrease in all-cause and non-CVD mortality (of 22% and 30%, respectively); however, no statistically significant association with short-term mortality (4 years) was observed [48].

Beside the beneficial legacy effect of optimized control on lipid profile, long-term detrimental effects of a transient exposure to hypercholesterolemia have also been demonstrated. In a study in young adults born prematurely, endothelial function assessed by measurements of pulse wave velocity was compared between those who received intravenous lipids at early stage of life as part of parenteral nutrition and matched controls with equivalent perinatal characteristics who did not receive such supplements. Perinatal hyperlipemia secondary to intravenous lipids administration was associated with a significantly altered endothelial function compared to controls in adult life [49].

### 3.2. Trained Immunity: oxLDL “Memory”

As for metabolic memory, several in vitro studies evaluated the effects of transient exposure to lipoproteins on the vascular wall or its components, leading to the concept of trained immunity [50] resulting from the priming of different cell types with oxLDL (Figure 1).

Upon pre-incubation with oxLDL in vitro and further stimulation by a toll-like receptor (TLR) agonist, such as lipopolysaccharide (LPS) or lipopeptide PAM3cys4, endothelial cells expressed ICAM-1, VCAM-1, and E-selectin, which are adhesion molecules controlling rolling, adhesion, and transendothelial migration of leukocytes. In parallel, oxLDL priming also induced the enrichment of activating histone marks (H3K27ac and H3K4me3) on the ICAM-1 promoter [51]. In human coronary smooth muscle cells in vitro, oxLDL similarly induced a proinflammatory priming effect with a significant increase in IL6, IL8, and MCP1 production following restimulation with a TLR agonist. This increase was blocked by the inhibition of mTOR and TRL signaling pathways and by the histone methyltransferase inhibitor methylthioadenosine (MTA) [52]. Similar results were observed in isolated human monocytes pre-exposed to oxLDL in vitro: when primed by oxLDL (and not with non-oxidized LDL), subsequent stimulation with a TLR agonist, such as LPS, induced expression of several proatherogenic proteins (e.g., IL-6, TNF-α, MCP-1, and MMP 2 and 9), enhanced foam cells formation, and increased H3K4me3 in promoter region of *TNFα*, *IL-6*, *IL-18*, and *MCP-1* genes. Pretreatment of monocytes with the histone methyltransferase inhibitor MTA completely prevented the oxLDL-induced proinflammatory phenotype [53]. Another pathway involved in oxLDL priming in monocytes in vitro is the production of ROS through mTOR activation of NADPH oxidase: cytosolic and mitochondrial ROS production was induced by oxLDL priming in monocytes in culture. This increase was blocked by pharmacological inhibition of mTOR, and the development of the trained immunity phenotype was blocked by antioxidant treatment [8]. Priming with oxLDL also has a major effect on metabolism in vascular wall cellular components with an increase in glycolysis and lactate production [50,54]. In preclinical studies in patients with established atherosclerosis, circulating monocytes also exhibited a higher production of pro-inflammatory cytokines upon LPS stimulation than healthy controls, associated with epigenetic remodeling (lower H3K27me3 on TNF𝛼 promoter) and increased expression of rate-limiting enzymes of the glycolysis pathway and the pentose phosphate pathway. Interestingly, this pro-inflammatory phenotype was present only in patients with severe symptomatic coronary atherosclerosis and not in patients with mild asymptomatic carotid atherosclerosis [55]. More recently, a concomitant upregulation of glycolytic activity and oxygen consumption was observed in oxLDL-primed human primary monocytes. In healthy volunteers, the impact of genetic variation (SNPs) in glycolytic genes on the training capacity of monocytes was evaluated: variants of genes encoding the inducible PFK-2/FBPase isozyme 6-phosphofructo-2-kinase/fructose-2,6-biphosphatase 3 (PFKFB3) and phosphofructokinase (PFKP) were associated with the potentiation of TNF-α and IL-6 production upon priming with oxLDL. Subsequent functional validation with inhibitors of glycolytic metabolism revealed dose-dependent inhibition of trained immunity in vitro. In vivo, the administration of metformin, a modulator of glucose metabolism, abrogated the ability of human monocytes to mount a trained response to oxLDL ex vivo [54].

## 4. Hypertension

### 4.1. Legacy Effect of Optimized Blood Pressure Control

Similar legacy findings were observed with blood pressure control (Table 1). A meta-analysis of 18 randomized clinical trials using blood pressure-lowering medications (including 132,854 patients in total) showed lower mortality (by 16%) in the different intervention groups during the trial period. Mortality was also lower (by 15%) during the open-label follow-up phases, when all of the patients were advised to take the same therapy, and rates of receiving active therapy were similar in the two groups [56]. In the Systolic Hypertension in the Elderly Program (SHEP) trial, chlortalidone-based therapy resulted in a lower rate of cardiovascular events (strokes, MI, and heart failure) at 4.5 years, but no significant effect on all-cause and cardiovascular mortality [57]. In the long-term follow-up of 22 years, a significant life expectancy gain, free from CVD-related death, was observed in the intervention group, corresponding to 1 day of life gained for each month of treatment [58]. In the Randomized Olmesartan and Diabetes Microalbuminuria Prevention (ROADMAP) trial, patients with type 2 diabetes with at least one other cardiovascular risk factor were assigned to receive either olmesartan or placebo for almost 3 years, with a principal favorable outcome of significantly delayed microalbuminuria onset in the intervention group [59]. In the observational follow-up of 3.3 years, despite an increase in mean systolic blood pressure in both groups, the incidences of diabetic retinopathy and congestive heart failure were significantly lower (OR 0.34 and 0.23, respectively) in the original intervention group. Moreover, patients who developed microalbuminuria during the trial had a higher incidence of cardio- and cerebrovascular events (OR 1.77) [60].

The above-mentioned ASCOT study also included one arm of blood pressure control, in which patients were assigned either to an amlodipine-based regimen (with perindopril if required) or to atenolol (with bendroflumethiazide if required). Although mean blood pressure reduction was similar in both groups, cardiovascular events and all-cause mortality was significantly lower in the amlodipine (plus perindopril) group, suggesting that hypotensive medications are not equal in the prevention of CVD [61]. Long-term follow-up to 16 years showed no overall difference in all-cause mortality but significantly fewer deaths from stroke (by 29%) in the amlodipine (plus perindopril) group [62]. The UKPDS trial also included a subgroup of newly diagnosed type 2 diabetic patients in which blood pressure control was optimized. Significant relative risk reductions were found during the trial for any diabetes-related end point, diabetes-related death, microvascular disease, and stroke in the group receiving tight compared to less-tight blood pressure control [63]. However, this effect was not sustained during the post-trial follow-up. Nevertheless, a risk reduction for peripheral vascular disease associated with tight blood pressure control remained significant after 10 years [64]. As previously mentioned, the HOPE-3 study included an arm to compare candesartan plus hydrochlorothiazide in intermediate-risk patients. No effect on both composite outcomes (MACE-1 and MACE-2) emerged after the intervention period, except in a subgroup of patients with high systolic blood pressure (>143.5 mmHg), with a significant decrease of 27% and 24%, respectively [65]. Long-term follow-up analysis revealed a legacy effect of combined therapy in the same subgroup of patients (>143.5 mmHg) with a significant decrease (of 24%) in MACE-1 composite outcome [44].

Other randomized studies, however, failed to detect any legacy effect in blood pressure lowering. In the previously cited study ALLHAT, more than 30,000 patients were randomized to receive either chlorthalidone, amlodipine, or lisinopril for at least 4 years (active phase) with an observational period of 8 to 13 years afterward. No differential effect between these interventions was observed during the trial phase, explaining the absence of legacy effect [66]. As cited above, the Second Australian National BP Study (ANBP2) compared elderly patients in two different blood pressure-lowering regimens (perindopril vs. diuretics). A benefit of ACE inhibition on cardiovascular events and deaths was observed, particularly in men [67]. Long-term observational post hoc analysis failed to identified any legacy effect, when “treatment naïve” patients were compared to those treated at inclusion [68]. This result could be explained by one of the main limitations of this study, i.e., a non-randomized post hoc analysis performed in a cohort of older patients (mean age 71 years). In a meta-analysis of three randomized studies, including close to 5000 moderately hypertensive middle-aged patients, early initiation of antihypertensive treatment did not reduce cardiovascular morbidity or mortality compared to delayed initiation. No legacy effect was therefore observed in the long-term follow-up observational post hoc analysis [69].

Nevertheless, one class of blood pressure-lowering therapy emerged from clinical studies with a potential beneficial effect on cardiovascular outcomes beyond blood pressure lowering. In patients with heart failure resulting from myocardial infarction, a RAAS inhibitor led to prognosis improvement with only minimal (if any) effect on blood pressure [70]. Moreover, numerous studies in diabetic nephropathy demonstrated renal protection by RAAS inhibition independent from its blood pressure-lowering effect. Further mechanistic studies led to the proposition that the reno-protection results from a decrease in angiotensin II-dependent glomerular efferent arteriolar tone, thereby reducing filtration pressure [70]. Finally, in the Losartan Intervention For Endpoint reduction in hypertension (LIFE) study, the AT1R blocking, RAAS inhibitor prevented more cardiovascular morbidity and deaths compared to atenolol, despite similar blood pressure reduction [71].

### 4.2. Ang II “Memory”

Angiotensin II, as the main product and effector of the RAAS stimulation, is a physiological regulator of blood pressure [72,73]. Infusions of Ang II are widely used experimentally to induce endothelial dysfunction or to mimic a hypertensive condition in vivo [74,75]. Contrary to the metabolic memory, published evidence on a putative memory effect of Ang II is scarce (Figure 1), aside from indirect causality inferred from the clinical trials with RAAS blockers, as reviewed above. A residual effect of temporary Ang II infusion on blood pressure and insulin sensitivity was observed in a study on young rats. A month after the end of infusion of a hypertensive dose of Ang II, blood pressure remained higher and insulin sensitivity was decreased in previously treated rats compared to saline-infused controls. These residual effects were attenuated by the co-administration of tempol, a free radical scavenger, or of candesartan together with Ang II during the infusion period, and the effects of candesartan were not mimicked by hydralazine at a dose producing a similar decrease in blood pressure; the data suggested a link with oxidative stress and an Ang II receptor specificity for this effect [76]. In another study on mice, a sustained vascular and heart injury was observed up to 1 week after withdrawal of an initial Ang II infusion with persistent activation of multiple signaling pathways (JNK1/2, STAT3, and NF-κB) and an increase in ROS production; as the sustained effect was attenuated with apocynin, a NOX inhibitor, the data suggested a link with persistent NADPH oxidase activation. However, these studies did not investigate the upstream mechanism for the sustained oxidative stress, including through potential epigenetic regulation [77,78]. In the above-mentioned model, hypertension persisted during the week after Ang II withdrawal, introducing a confounding bias about its role in the persisting cardiac and vascular remodeling. More recently, we observed long-term (up to 3 weeks) detrimental effects of temporary Ang II infusion on heart and vascular integrity, linked to a sustained vascular smooth muscle cell phenotypic switch and down-regulation of 𝛼-smooth muscle actin, associated with epigenetic marks (H3K27me3) and myocardin transcription factor repression [Pothen L et al.; unpublished data].

## 5. Discussion

This review of clinical trials testing optimized control of cardiovascular risk factors in at-risk patients provided convincing evidence in favor of a legacy effect, despite heterogeneity in study populations, duration of in-trial and follow-up periods, and clinical outcomes. A legacy effect was firmly established in diabetic patients, but also discernible in hypercholesterolemic and hypertensive patients. One common feature is that, to be effective and produce legacy, interventions on risk factors have to be implemented at early stages. The expression “the sooner the better” should therefore be extended from diabetes to the control of hypertension and hyperlipidemia.

The mechanisms underlying such legacy effects were thoroughly examined in diabetic conditions with preclinical models showing metabolic changes that led to sustained oxidative stress and epigenetic modifications; these, in turn, induced a feed-forward phenomenon (or “vicious circle”) that perpetuated oxidative stress, inflammation, and damage to the vascular wall [7]. The mechanisms for trained immunity by exposure to oxLDL [50] or Ang II memory [76,77] [Pothen L et al., unpublished] are less well-characterized, but probably converge on, at least, some common effectors.

Even if pathophysiological mechanisms are not completely understood, the idea of an organ memory rather than memory specific to each risk factor is an attractive theory, which needs to be substantiated by further investigation, either through clinical trials or in vitro/in vivo studies. Arterial beds, for example, can be considered as a unique organ, consisting of highly sensitive and interacting cell types, directly in contact with blood flow where the inner endothelial cell lining could perpetuate oxidative stress induced by an initial aggressor; among these, oxLDL is known to produce endothelial dysfunction and to prime monocytes, thereby promoting pro-inflammatory susceptibility [29,52]; in response, the underlying vascular smooth muscle cells can (reversibly) alternate between a contractile or synthetic phenotype with more cytokines and extracellular matrix production resulting in sustained vascular remodeling. As all aggressors converge on the same resulting phenotype (e.g., atherosclerosis) and as they cumulate their effects together—indeed hypertension, lipid disorders, and diabetes often coexist within the same individual—one can imagine that despite differences in the initial steps of the disease, all cardiovascular risk factors lead to the same memory behavior of the vascular wall, just as rivers meet in the same sea.

Notably, epigenetic mechanisms appeared at the center of all the above-mentioned memory effects. In metabolic memory, several epigenetic changes (histone methylation, histone, and protein acetylation, CpG island methylation content, miRNA) were involved in the sustained oxidative stress in endothelial cells [7,30]. In trained immunity, H3K4me3 and H3K27ac were identified as marks of priming with oxLDL in monocytes [50] and endothelial cells [51], associated with the subsequent pro-inflammatory phenotype. In Ang II memory, we observed another repressive epigenetic mark in vascular smooth muscle cells (K3K27me3), associated with sustained phenotypic switch and vascular damage [Pothen L et al., unpublished]. Despite the lack of congruency in all these epigenetic changes, one can recognize that, albeit different, they share common features and lead to persistent activation of common effectors, consistent with the idea of an organized memory pattern of the arterial bed.

## 6. Perspectives

Some of the epigenetic changes at the basis of a legacy effect of precited cardiovascular risk factors were also implicated more broadly in the development of cardiometabolic diseases.

In another model of hypertension in vivo, spontaneously hypertensive rats (SHR) exhibited a significantly higher expression of angiotensin 1α receptor (AT1aR), encoded by Atgr1α, compared to Wistar-Kyoto control rats (WKY), which was associated with an hypomethylation of the Atgr1α promoter [79]. In other studies, SHR rats also showed higher expression of Ace-1 mRNA and protein (compared to WKY controls) associated with H3 acetylation enrichment and H3K4me3 (activating marks) in Ace-1 promoter regions [79]. Insulin-resistant morbidly obese subjects have a different DNA methylation pattern in visceral adipose tissue, compared to insulin-sensitive ones, resulting in close to a 10% difference in diabetes-related expressed genes [80]. An epigenome-wide association study showed that elevated BMI was associated with changes in DNA methylation, mostly in genes involved in lipid and lipoprotein metabolism, substrate transport, and inflammatory pathways and that these alterations are related to adiposity [80]. Mice deficient in histone demethylase HDM2a, an enzyme responsible for H3K9 demethylation, developed an adult-onset obesity, hypertriglyceridemia, and hypercholesterolemia, as well as insulin resistance compared to wild type controls [81]. Sirtuins, a class of HDACs, were reported to act as metabolic regulators of glucose homeostasis, insulin resistance, and its associated inflammation. As an example, lack of SIRT1-, SIRT2-, and SIRT6-dependent deacetylation and activation of specific adipose gene programs was shown to contribute to the development of metabolic disorders, such as type 2 diabetes and obesity [81]. miRNA-mediated epigenetic changes also induce obesity-associated adipose tissue inflammation, a significant factor responsible for developing insulin resistance and type 2 diabetes. Accordingly, obese adipocyte-derived exosomes have an increased expression of miR-29a; when transferred into adipocytes, myocytes, and hepatocytes, these exosomes produced insulin resistance in both in vitro and in vivo models [81]. Of note, epigenetic changes are heritable and can be transmitted to the offspring. Transgenerational inheritance of specific epigenetic patterns was shown to affect expression of genes involved in vascular inflammation, oxidative stress, and atherosclerosis [80]

As epigenetic modifications are reversible, they could be interesting targets for innovative therapies. Histone deacetylase (HDAC) inhibitors were tested in experimental diabetes with some favorable effect: vorinostat, a nonspecific HDAC inhibitor, showed beneficial effects on diabetic nephropathy in mice [82]. An agonist of SIRT1, a class III HDAC that suppresses AGE-induced expression of profibrotic genes through an antioxidant effect, exerted a favorable effect on kidney injury in diabetic mice [83]. Inhibitors of histone methyltransferases were also studied [84]. Finally, siRNAs, antagomirs, or antisense oligonucleotides targeting epigenetic modifiers and lncRNAs/miRNAs demonstrated promising results [85]. Further studies will definitively be needed to design more specific inhibitors (HDAC-HMT) and to decrease off-target effects before evaluating their efficacy in the clinic.

## 7. Conclusions

In conclusion, clinical trials on the optimization of cardiovascular risk factors demonstrated the legacy effect of interventions on glycemic, lipid profile, or blood pressure control. While the mechanisms underlying this organ legacy are not yet fully understood, future investigations are expected to identify epigenetic signatures of “memory” that facilitate early detection of patients at risk and, possibly, lead to new therapeutic strategies by erasing them.

## Figures and Tables

**Table 1:** Studies evaluating legacy effect in cardiovascular risk factors control.

	Study Name *Type*	Patients Characteristics (n int/n Follow-Up)	Effect of Intervention (Study Duration)	Legacy Effect (Follow-Up Duration)	Comments
***Diabetes***	**DCCT** *RCT* **EDIC** *OFU*	T1DM Mean age 27 y (1441/1394)	(6.5 y) (−) 53% severe retinopathy (−) 40% clinical neuropathy (−) 61 % microalbuminuria	(17 y) Legacy effect of intervention (−) 57% nonfatal MI, stroke, or death from CVD (−) 46% nephropathy	In OFU Hb1Ac 7.9% intensive vs. 7.8% conventional
	**UKPDS** *RCT/OFU*	Newly diagnosed T2DM Mean age 56.4 y (3867/3277)	(10 y) (−) 12% diabetes-related endpoints (−) 10% diabetes-related death (−) 25% microvascular endpoints	(10 y) Legacy effect of intervention (−) 9% diabetes-related endpoints (−) 24% microvascular endpoints (−) 15% MI (−) 13% death from any cause	No difference in Hb1Ac in OFU
	**VADT** *RC/OFU*	T2DM long-duration (11.5 y) poorly controlled Mean age 60.4y (1791/1391)	(5.6 y) No difference in major CV outcomes, death, or microvascular complications except for progression of albuminuria (13.1% vs. 9.1% in int. treat. group)	(10 y) Legacy effect of intervention (−) 17% major CV events (15 y) No difference in major CV events or death	40% already had CV event at inclusion HbA1c curves still separated at 10 y follow-up, no more at 15 y
	**ACCORD** *RCT* **ACCORDION** *OFU*	T2DM long-duration (10 y) Mean age 62.2 y (10,251/8601)	(3.5 y) No effect on primary composite endpoint (non-fatal MI, non-fatal stroke, or death) (−) 24% MI in int. treat. group (+) 22% death from any cause (+) 36% death from CV causes in int. treat group	(9 y) No effect on primary composite endpoint Trend to lower non-fatal MI (+) 20% death from CV causes in int. group	35% had previous CV event
	**ADVANCE** *RCT* **ADVANCE-ON** *OFU*	T2DM long-duration (8 y) ≥55 y ≥1 CV risk factor or history of major macro- or microvascular disease (11,140/8494)	(5 y) (−) 10% combined major micro- and macrovascular events (−) 21% nephropathy No effect on major macrovascular events, CV death, or death from any cause	(6 y) No differences in risk of death or major CV events (−) 46% end stage renal disease but very few events	
	**ADDITION** *Registered based non-RCT*	Newly diagnosed T2DM after screening compared to unscreened population Mean age 59.9 y (registered 153,107, diagnosed 1533)	(5 y screening period) No comparison in intervention Diabetes detected 2.2 y earlier in screened group	(10 y) In screened group: HR 0.79 lower mortality HR 0.80 lower CV mortality HR 0.66 lower diabetes-related mortality HR 0.84 lower CVD event	No effect at national population level
	**DAS** *Cohort study*	Newly diagnosed T2DM Mean age 56.8 y Stratified by mean Hb1Ac during first year, comparison to Hb1Ac < 6.5% (34,737)	NA	(10 y) - Hb1Ac ≥ 6.5% within first year: increased micro- and macro-CV events (HR 1.2) - Hb1Ac ≥ 7.0% within first year, increased mortality (HR 1.29) - ≥8.0% for more than 2 y increased microvascular event and mortality risk	
***Lipid profile***	**LIPID** *RCT* **LIPID FU** *OFU*	Pravastatin vs. placebo in recent MI or unstable angina Median age 62 y Median cholesterol 218 mg/dl (9014/7680)	(6 y) Vs. placebo: (−) 24% RR death from CHD (−) 22% RR overall mortality (−) 29% RR MI (−)19% RR stroke	(2 y) Open-label period, crossover 86% gr. placebo on pravastatin 88% gr. prava still on pravastatin, with similar cholesterol level Legacy effect of intervention (−) 25% RR death from CHD (−) 19% RR overall mortality (−)15% RR MI (−) 24% RR stroke	
	**ASCOT-LLA** *RCT* **UK ASCOT-LLA** **legacy** *OFU*	Atorvastatin vs. placebo in hypertensive patients with at least 3 other CV risk factors in primary prevention Mean age 61.4 y (4605/4432)	(3.3 y) Favor atorvastatin HR 0.64 for non-fatal MI and fatal CHD HR 0.79 for CV events HR 0.71 for coronary events Trend to less death (HR 0.87, *p* = 0.16)	(11 y) Open-label crossover (2/3 placebo on statin) Legacy effect favored atorvastatin HR 0.86 all-cause mortality HR 0.85 non-CV death HR 0.89 CV death but not significant	
	**WOSCOPS** *RCT* **WOSCOPS FU** *OFU*	Pravastatin vs. placebo in primary prevention in men Mean age 55 y High LDL (6596/6408)	(4.9 y) Favor pravastatin: (−) 31% RR in non-fatal MI or death from CHD (−) 32% RR in death from CV causes	(18 y) 38.7% (former prava) and 35.2% (former placebo) at 5 y on statin, no further data Legacy effect favored pravastatin: (−) 13% mortality (−) 21% RR in death from CV causes	
	**ACCORD-LLA** *RCT* **ACCORDION** *OFU*	Combined therapy (simvastatin + fibrate vs. simva+ placebo) in T2D long-duration with dyslipidemia Mean age 61.8 y (940/765)	(5 y) No effect of combined treatment on CV outcomes or mortality	(10 y) Same level of lipid profile in both groups but legacy effect of combined treatment HR 0.68 in all-cause mortality HR 0.63 in CVD mortality HR 0.66 in major CHD	
	**HOPE-3** *RCT* **HOPE-3 FU** *OFU*	Rosuvastatin vs. placebo, at least 1 CV risk factor, no CV disease Mean age 65.7 y (12,705/9326)	(5.6) Favor rosuvastatin HR 0.76 in MACE-1 (composite of death from CV causes, non-fatal MI, non-fatal stroke) HR 0.75 in MACE2 (composite of revascularization, HF, resuscitated CA)	(3.1) Legacy effect of rosuvastatin treatment HR 0.80 MACE-1 HR 0.83 MACE -2 Total FU: HR 0.79 MACE-1 HR 0.79 MACE -2	In OFU: 37% on statin (36% of former rosu, 38% of former placebo group)
	**ALLHAT-LLT** *RCT* **ALLHAT-LLT** *OFU*	Pravastatin vs. usual care in hypertensive patient + at least 1 CV risk factor Mean age 66 y (10,355/1672)	(4.8 y) No effect on all-cause mortality or CHD	(8–13 y) No legacy effect	Only 16% difference in LDL between groups at end of intervention period
***Blood pressure***	**SHEP** *RCT* **SHEP FU** *OFU*	Chlortalidone +/− atenolol vs. placebo in isolated hypertensive patients Mean age 71.6 y (4736/1885)	(4.5 y) Favor intervention 0.64 RR in stroke 0.73 RR in non-fatal MI + 0.46 RR LV failure	(22 y) Legacy effect of intervention HR 0.89 CV death One day of life expectancy gained in intervention group per month of treatment	
	**ROADMAP** *RCT* **ROADMAP OFU** *OFU*	Olmesartan vs. placebo in T2D patients ≥ 1 CV risk factor Mean age 57.7 y (4447/1758)	(3.2 y) Increased time to onset of microalbuminuria (25%)	(6 y) Despite crossover and increase in BP, legacy effect of olmesartan OR 0.34 in diabetic retinopathy OR 0.23 in CHF	! higher rate of fatal CV events in int. group in patients with pre-existing CHD
	**ASCOT** *RCT* **ASCOT legacy** *OFU*	Amlodipine (+/− perindopril) vs. atenolol (+/− thiazide) hypertensive patients ≥ 3 other CV risk factors Mean age 63 y (19,257/8580)	(5.5 y) Favor amlodipine-based regiment HR 0.77 stroke (fatal and non-fatal) HR 0.84 in CV events HR 0.76 in CV mortality HR 0.70 in new onset of diabetes	(16 y) Legacy effect of amlodipine-based regiment HR 0.71 death from stroke	
	**UKPDS 38** *RCT* **FU** *OFU*	Tight vs. less-tight blood pressure control T2DM patients (captopril and atenolol) Mean age 56.8 y (1148/884 )	(8.4 y) Favor tight control (−) 24% diabetes-related endpoints (−) 32% diabetes-related deaths (−) 44% strokes (−) 37% microvascular endpoints	(10 y) Legacy effect favored tight control 0.50 RR in peripheral vascular disease	
	**HOPE-3** *RCT* **HOPE-3 FU** OFU	Candesartan + HCTZ vs. placebo at least 1 CV risk factor No CV disease Mean age 65.7 y (12,705/9326)	(5.6 y) No significative difference, except in subgroup > 143.5 mmHg HR 0.73 MACE 1 HR 0.76 MACE 2	(3.1 y) Legacy effect in subgroup > 143.5 mmHg HR 0.76 MACE 1	2/3 in follow-up 1 ≥ BP-lowering drug and 30% on 2 ≥ BP-lowering drugs (similar in both groups)
	**ALLHAT** *RCT*	Hypertensive patients+ ≥1 CHD risk factor, chlortalidone vs. amlodipine vs. lisinopril Mean age 67 y (32,804/27,755)	(4.9 y) No difference Increase in HF for amlodipine Increase in stroke mortality for lisinopril	(8–13 y) No legacy effect	
	**ANBP2** *RCT* **ANBP2 FU** *OFU*	Hypertensive patients ACE inhibitor vs. diuretics Mean age 72 y (6083/5378)	(4.1 y) Favor ACE inhibition HR 0.89 CV event or death HR 0.68 nonfatal MI but HR 1.91 for fatal stroke	(10 y) No legacy effect	Different comparison in OFU: treatment-naïve vs. not naïve

Abbreviations: T1DM: type 1 diabetes mellitus; T2DM: type 2 diabetes mellitus; CV: cardiovascular; MI: myocardial infarction; y: years; RCT: randomized control trial; CHD: coronary heart disease; CHF: congestive heart failure; HR: hazard ratio; RR: relative risk; OR: odd ratio; FU: follow-up; OFU: observational follow-up.
